# Supplementary material for: Prospect theory, constant relative risk aversion, and the investment horizon
Source: PLoS One. 2021 Apr 1;16(4):e0248904. doi: 10.1371/journal.pone.0248904 (PMC8016345; doi:10.1371/journal.pone.0248904)
Supplement: S1 Appendix — (DOCX) [file pone.0248904.s001.docx]

**S1 Appendix: Optimal Asset Allocation for a PT Investor with Alternative Reference Points**

**Case 2: Reference point at Current Wealth**

In this case we also obtain a discontinuous jump in the optimal investment proportion in the stock. The difference relative to Case 1 (described in the text) is that the jump is not from 0 to 1, but rather from some positive value (about 1/3 for the distributions given in the experiment) to 1. Again, this result is general, and does not depend on the specific return distribution. In what follows we will first provide the general analysis, and then discuss the specific tasks used in the experiment.

As in Case 1, the terminal wealth is given by: . However, the change of wealth is calculated relative to current wealth, $W_{0}$, i.e.:

. (A1)

We denote the excess return on the stock by and $r_{f}\equiv R_{f}-1$. Without loss of generality, we take $W_{0}=1$. Then, we have:

. (A2)

We assume that the expected excess return is positive $E\left[\tilde{r}\right][]>0$ and that the interest rate is positive$r_{f}>0$. Thus, if *r* is positive, then the change of wealth, *x,* is positive for any $1\geq w\geq0$. If *r* is negative, *x* can be either positive or negative, depending on *w*. Let us denote the minimum value of $\tilde{r}$ by $r_{\min}$. In any non-degenerate situation we have $r_{\min}<0$ (if $r_{\min}>0$, the minimum return on the stock is higher than the risk-free return, and the stock thus dominates the risk-free asset by First-order Stochastic Dominance). $r_{\min}$ is also bounded from below, because the rate of return on the stock can’t be lower than -1 (-100%).

From eq.(A2) we conclude that if $w<-\frac{r_{f}}{r_{\min}}$ then $\tilde{x}$ is positive for any value of $\tilde{r}$, and we have:

$EV=\int_{r_{\min}}^{\infty} \left(wr+r_{f}\right)^{\alpha}f(r) dr$. (A3)

If $w>-\frac{r_{f}}{r_{\min}}$ then $\tilde{x}$can be either negative or positive. Specifically, it is negative for $r<-\frac{r_{f}}{w}$, and it is positive for $r>-\frac{r_{f}}{w}$(see eq.(A2)). Thus, in the range $w>-\frac{r_{f}}{r_{\min}}$we have:

$EV=-\lambda\int_{r_{\min}}^{-\frac{r_{f}}{w}} \left(-\left(wr+r_{f}\right)\right)^{\alpha}f(r) dr +\int_{-\frac{r_{f}}{w}}^{\infty} \left(wr+r_{f}\right)^{\alpha}f(r) dr$. (A4)

The PT expected value given by eqs.(A3) and (A4) is continuous across the two ranges, but its derivative with respect to *w* is not. Let us analyze each of these ranges separately.

**Range 1 $w<-\frac{r_{f}}{r_{\min}}$:**

The first and second derivatives of the *EV* in eq.(A3) with respect to the investment proportion, *w*, are: (A5)

$\frac{\partial EV}{\partial w}=\alpha\int_{r_{\min}}^{\infty} \left(wr+r_{f}\right)^{\alpha-1}r f(r) dr$ and $\frac{\partial^{2}EV}{\partial w^{2}}=\alpha\left(\alpha-1\right)\int_{r_{\min}}^{\infty} \left(wr+r_{f}\right)^{\alpha-2}r^{2} f(r) dr$.

The first derivative may generally be either positive or negative, because *r* is negative in the range $\left[r_{\min}, 0\right]$ and positive in the range [0, $\infty].$ The second derivative is negative: notice that the integral in the second derivative is positive (because $wr+r_{f}>0$), and the PT risk-aversion in the domain of gains implies $0<\alpha<1$. Also, the first derivative at *w*=0 is positive (because the expected value of *r* is positive). Thus, in the range $\left[0, -\frac{r_{f}}{r_{\min}}\right]$ the maximum value of *EV* is obtained either at some internal point $0<w<-r_{f}/r_{\min}$, or at the end point $w=-r_{f}/r_{\min}$. Notice that the existence of a maximum point in the range $\left[0, -\frac{r_{f}}{r_{\min}}\right]$ is independent of the distribution$f(r)$, i.e. of the investment horizon, and of the preference parameters (although the exact location of the maximum point does depend on these factors).

When we consider the whole range of the investment weight in the stock, $1\geq w\geq0$, the above maximum point may be either the global maximum or a local maximum, depending on the behavior of the *EV* at values of *w* exceeding $-r_{f}/r_{\min}$: if the *EV* decreases in the range $w>-r_{f}/r_{\min}$, then the above maximum point is global. However, if the *EV* increases in the range $w>-r_{f}/r_{\min}$, the global maximum may be in found in this second range. Thus, we turn next to analyze the *EV* in the range $w>-r_{f}/r_{\min}$, namely, in the general case where the change of wealth, *x*, may be either negative or positive.

**Range 2 $w>-\frac{r_{f}}{r_{\min}}$:**

In this range, the derivative of the *EV* in eq.(A4) with respect to *w* is given by[[1]](#footnote-1):

$\frac{\partial EV}{\partial w}=-\lambda\alpha\int_{r_{\min}}^{-\frac{r_{f}}{w}} \left(-\left(wr+r_{f}\right)\right)^{\alpha-1}\left(-r\right)f(r) dr +\alpha\int_{-\frac{r_{f}}{w}}^{\infty} \left(wr+r_{f}\right)^{\alpha-1}r f(r) dr$. (A6)

It is convenient to divide the second integral to its positive and negative parts: (A7)

$\frac{\partial EV}{\partial w}=-\lambda\alpha\int_{r_{\min}}^{-\frac{r_{f}}{w}} \left(-\left(wr+r_{f}\right)\right)^{\alpha-1}\left(-r\right)f(r) dr +\alpha\int_{-\frac{r_{f}}{w}}^{0} \left(wr+r_{f}\right)^{\alpha-1}r f(r) dr+\alpha\int_{0}^{\infty} \left(wr+r_{f}\right)^{\alpha-1}r f(r) dr$The first two terms above are negative, while the third is positive: in the first term both $-\left(wr+r_{f}\right)$ and –*r* are positive and we have a minus sign in front of the integral, hence the first term is negative; in the second and third terms $wr+r_{f}$is positive, while *r* is negative in the second term, but is positive in the third term. The overall sign of the derivative in eq.(A7) depends on the relative magnitudes of these three terms.

For short horizons the probability that the excess return *r* is negative is typically substantial. Thus, the first two terms, which are integrals over *r* in the range $\left[r_{\min}, 0\right]$ are relatively large, because of the overweighing of the first term by the loss aversion factor $\lambda$, and the overall derivative may be negative. Thus, for short horizons the *EV* my decrease in the range $w>-r_{f}/r_{\min}$, implying that the maximum *EV* is obtained in the range $0<w\leq-r_{f}/r_{\min}$. However, as the horizon increases, the probability that the excess return is negative decreases to 0, and the first two terms in eq.(A7) become smaller, while the third term becomes larger. Thus, as the horizon increases the derivative (A7) becomes positive, for any value of $w>-r_{f}/r_{\min}$, and the optimum investment weight jumps from somewhere in the range $0<w*\leq-r_{f}/r_{\min}$ to $w*=1$.[[2]](#footnote-2)

Thus, like in Case 1, with the reference point at the future value of wealth invested in the risk-free asset, we again obtain a jump in the investment weight in the risky asset, albeit not from zero to 1 but from some positive value smaller than 1 to 1. Before discussing the optimal asset allocation in the tasks employed in our experiment, let us first take a short detour to address the following question: From a practical/empirical perspective, how long does the horizon have to be for the jump to occur? After all, if the jump occurs at a horizon of 100 years, this is not of much practical interest. In order to answer this question, we perform the following analysis employing the empirical return distributions. We consider the optimal asset allocation between the S&P500 index and 3-month T-bills. Figure A1 shows the optimal proportion in the index as a function of the investment horizon, for various PT parameters. The T-year return distribution for the stock index is estimated by randomly drawing 50,000 sets of T returns (with replacement) from the set of annual returns on the S&P500 index during 1997-2016. The T-bill rate is taken as the average annual rate over this period (2.14%). For a PT investor with λ=3 and α=0.88 the optimal investment proportion in the stock index is 9% if the horizon is 1 year, and it jumps to 100% if the horizon is 2 years or longer. (For a PT investor with the Tversky and Kahneman (1992) parameters of with λ=2.25 and α=0.88, which is less loss-averse, the optimal proportion in stocks is 100% already at the 1-year horizon, and remains 100% at all horizons). For an investor with λ=3 and α=0.6 the optimal investment proportion in the stock index is 5% if the horizon is 1 year, 13% for 2 years, 27% for 3 years, and it then jumps to 100%. Similarly, other PT preference parameters also yield dramatic jumps in the optimal allocation. Figure A1 reveals that the jump typically occurs at very short horizons of 2-3 years. Even with rather extreme loss aversion, λ=5, and high risk aversion, α=0.6, a PT investor with a horizon longer than 6 years should invest 100% in stocks (and if borrowing is possible, up to the maximal leveraged position possible).

**
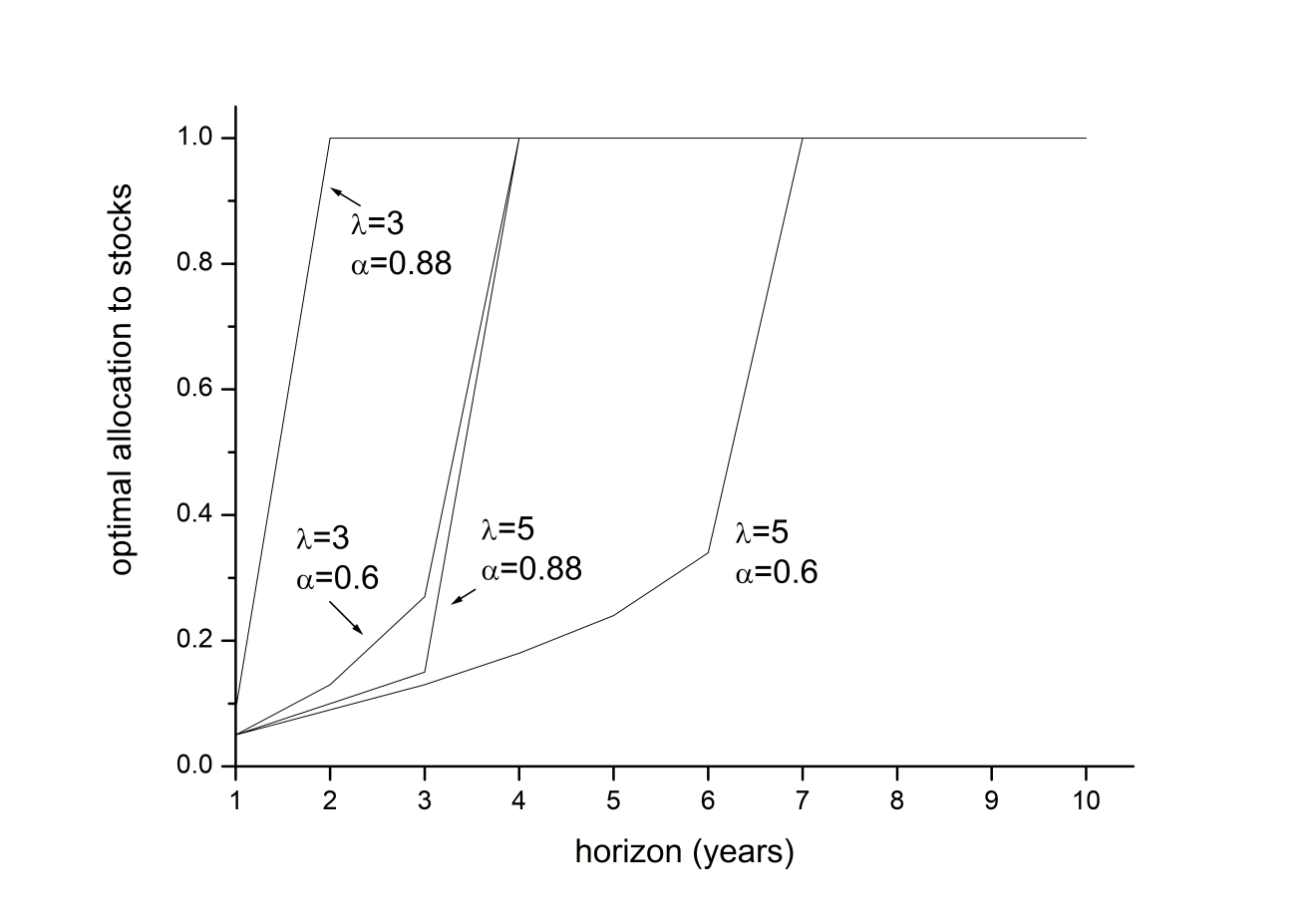
**

**Figure A1**: The optimal asset allocation between the S&P500 index and 3-month T-bills for PT investors as a function of the investment horizon. We employ the empirical S&P500 returns during 1997-2016 to construct the return distributions at various horizons. We construct the T-year return distribution by randomly drawing 50,000 sets of T returns (with replacement) from the set of annual returns on the S&P500 index during 1997-2016. The T-bill rate is taken as the average annual rate over this period (2.14%). The optimal allocation to the stock typically grows gradually with the horizon to a value in the range 10%-30%, and then “jumps” dramatically to 100%.

We should note that this dramatic jump in the asset allocation contrasts the common practice of the widely popular life cycle mutual funds. These funds *gradually* decrease the proportion allocated to risky assets every few years, as the savers become older, and the expected horizon shortens. In contrast, as Figure A1 shows, for the case where the reference point is at the current wealth, PT advocates an allocation of 100% in stocks for all but the shortest horizons, and then a sharp decrease to an allocation of only 10%-30% in stocks for very short horizons (or to 0% if the reference point is at the future value of wealth).

Let us now return to the specific prospects employed in our experiment. In Task 1 the stock yields either *R*=1.3 or *R*=0.9 with equal probabilities. If *R*=1.3, we have:

$x=\left[w\left(1.3-1.05\right)+1.05-1\right]=\left[0.25\cdot w+0.05\right]$, (A8)

and if *R*=0.9 we have:

$x=\left[w\left(0.9-1.05\right)+1.05-1\right]=\left[-0.15\cdot w+0.05\right]$. (A9)

(Recall that without loss of generality we take the initial wealth to be $W_{0}=1$). While the change of wealth in eq.(A8) is positive for any *w*, the sign of the change of wealth in eq.(A9) depends on *w*: this expression is positive (i.e. it is considered as a gain) if *w*<1/3, however, if *w*>1/3, *x* is negative (i.e. a loss). Thus, we can write the EV in Task 1 as:

$EV(w)=\left\{\begin{matrix} \frac{1}{2}\left[\left(0.25w+0.05\right)^{\alpha}+\left(-0.15w+0.05\right)^{\alpha}\right] if w\leq1/3 \\ \\ \frac{1}{2}\left[\left(0.25w+0.05\right)^{\alpha}-\lambda\left(0.15w-0.05\right)^{\alpha}\right] if w>1/3 \end{matrix}\right.$. (A10)

To see that this expression implies corner solutions, it is instructive to first concentrate on the simplified case of a piecewise linear value function where $\alpha=1$ (as discussed by Benartzi and Thaler 1995). In this case, plugging $\alpha=1$ simplifies eq.(A10) to:

$EV(w)=\left\{\begin{matrix} \frac{1}{2}\left[0.1\cdot w+0.1\right] if w\leq1/3 \\ \\ \frac{1}{2}\left[\left(0.25-0.15\lambda\right)w+0.05\left(1+\lambda\right)\right] if w>1/3 \end{matrix}\right.$. (A11)

As in the general case, the *EV* is continuous in *w*, but its derivative is not. Figure A2 plots the *EV* in eq.(A11) as a function of *w*. The *EV* increases linearly up to the point *w*=1/3. For *w*>1/3 the *EV* either continues to increase or starts to decrease, depending on the sign of the expression 0.25-0.15λ. If λ<0.25/0.15=1.66, this expression is positive and the *EV* continues to increase linearly in the range *w*>1/3, implying that the optimal investment proportion in the stock is 1. If λ>1.66 this expression is negative, implying that the *EV* decreases linearly in the range *w*>1/3, and thus that the maximum *EV* is obtained at *w*=1/3.


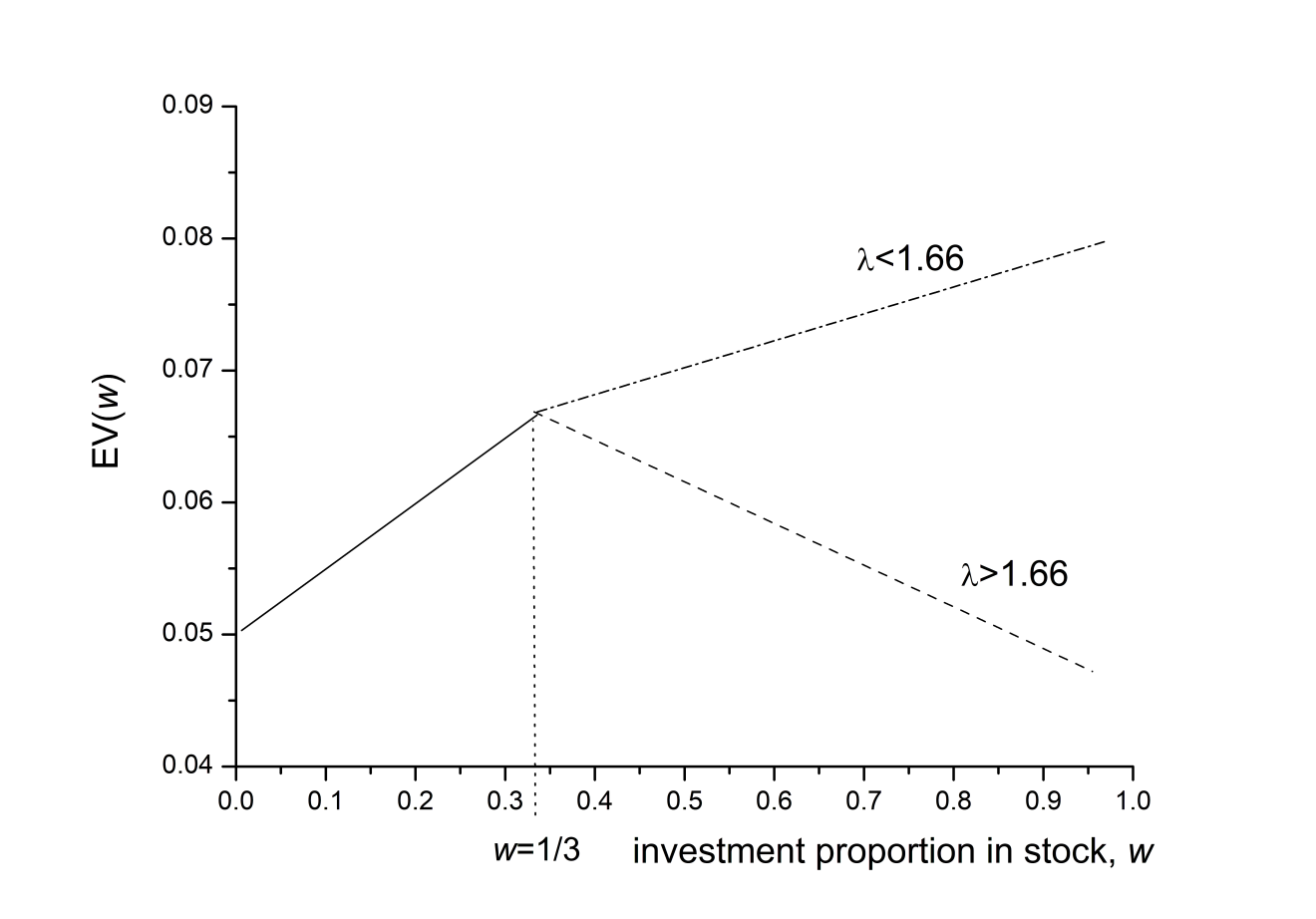


**Figure A2**: The PT expected value as a function of the investment proportion in the stock in Task 1. The reference point is the current wealth, and a piecewise linear value function is employed ($\alpha=1$). There are two possible optimal allocations: *w*=1/3 for λ>1.66, and *w*=1 for λ<1.66.

When $\alpha\neq1$ the results are similar, but as the *EV* is not linear in *w*, the maximum EV may be obtained at values lower than 1/3. The derivative of the *EV* with respect to *w* in the range *w*<1/3 is:

$\frac{\partial EV}{\partial w}=0.25\alpha\left(0.25w+0.05\right)^{\alpha-1}-0.15\alpha\left(-0.15w+0.05\right)^{\alpha-1}$, (A12)

and the maximum EV in this range is obtained at[[3]](#footnote-3):

$w*=\frac{0.05\cdot\left(\frac{3}{5}\right)^{\frac{1}{\alpha-1}} - 0.05}{0.15\cdot\left(\frac{3}{5}\right)^{\frac{1}{\alpha-1}} + 0.25}$. (A13)

In the range *w*>1/3 the derivative is either positive for all *w*, or negative for all *w*, depending on the values of $\alpha$ and $\lambda$.[[4]](#footnote-4) Thus, the optimal proportion is either given by eq.(A13), or is 1.

For $\alpha=0.88$ the optimal investment proportion in the range *w*<1/3 is obtained at:

$w*=\frac{0.05\cdot\left(\frac{3}{5}\right)^{\frac{1}{0.88-1}} - 0.05}{0.15\cdot\left(\frac{3}{5}\right)^{\frac{1}{0.88-1}} + 0.25}=0.32$. (A14)

Thus, for $\alpha=0.88$ there are two possible solutions in Task 1: either 0.32 or 1, depending on the value of $\lambda$. Panel A of Table AI provides the optimal asset allocation in Task 1 for various combinations of $\alpha$ and $\lambda$, when the reference point is taken as current wealth. Panels B and C provide the corresponding optimal asset allocations in Tasks 2 and 3 (calculated numerically).

Comparing the three panels of Table AI, corresponding to the case of a reference point at current wealth, reveals similar “jump” pattern in the optimal asset allocation as in Case 1, where the reference point is at the future value of wealth. As the horizon increases, the optimal proportion in the stock jumps dramatically from values of about 1/3 to 1. For example, for the Tversky and Kahneman (1992) parameters of $\alpha=0.88 \lambda=2.25$ the optimal investment proportion in the stock increases from 0.32 in Task 1 to 0.35 in Task 2, and then jumps to 1 in Task 3. The results are similar when CPT decision weights are employed instead of the objective probabilities, as shown in table A2.

To summarize Case 2, with a reference point at current wealth, we prove that in the general case there is a jump in the PT optimal allocation to the risky asset as the horizon increases. The jump is from some value smaller than (or equal to) $-r_{f}/r_{min}$ for short horizons to 1 (or more, if borrowing is possible) for longer horizons.

**Table AI**

Optimal Investment Proportion in the tock in the three tasks for PT Preferences with Reference Point at the Current Wealth ($W_{0}$). The highlighted cells correspond to the Tversky and Kahneman (1992) parameters of $\alpha=\beta=0.88 \lambda=2.25$. Objective probabilities are employed.

**A: Task 1**

| Lambda\alpha | 1.00 | 0.98 | 0.96 | 0.94 | 0.92 | 0.90 | 0.88 | 0.86 | 0.84 |
| --- | --- | --- | --- | --- | --- | --- | --- | --- | --- |
| 1.00 | 1.00 | 1.00 | 1.00 | 1.00 | 1.00 | 1.00 | 1.00 | 1.00 | 1.00 |
| 1.25 | 1.00 | 1.00 | 1.00 | 1.00 | 1.00 | 1.00 | 1.00 | 1.00 | 0.30 |
| 1.50 | 1.00 | 1.00 | 1.00 | 0.33 | 0.33 | 0.33 | 0.32 | 0.31 | 0.30 |
| 1.75 | 0.33 | 0.33 | 0.33 | 0.33 | 0.33 | 0.33 | 0.32 | 0.31 | 0.30 |
| 2.00 | 0.33 | 0.33 | 0.33 | 0.33 | 0.33 | 0.33 | 0.32 | 0.31 | 0.30 |
| 2.25 | 0.33 | 0.33 | 0.33 | 0.33 | 0.33 | 0.33 | 0.32 | 0.31 | 0.30 |
| 2.50 | 0.33 | 0.33 | 0.33 | 0.33 | 0.33 | 0.33 | 0.32 | 0.31 | 0.30 |
| 2.75 | 0.33 | 0.33 | 0.33 | 0.33 | 0.33 | 0.33 | 0.32 | 0.31 | 0.30 |
| 3.00 | 0.33 | 0.33 | 0.33 | 0.33 | 0.33 | 0.33 | 0.32 | 0.31 | 0.30 |

**B: Task 2**

| Lambda\alpha | 1.00 | 0.98 | 0.96 | 0.94 | 0.92 | 0.90 | 0.88 | 0.86 | 0.84 |
| --- | --- | --- | --- | --- | --- | --- | --- | --- | --- |
| 1.00 | 1.00 | 1.00 | 1.00 | 1.00 | 1.00 | 1.00 | 1.00 | 1.00 | 1.00 |
| 1.25 | 1.00 | 1.00 | 1.00 | 1.00 | 1.00 | 1.00 | 1.00 | 1.00 | 1.00 |
| 1.50 | 1.00 | 1.00 | 1.00 | 1.00 | 1.00 | 1.00 | 1.00 | 1.00 | 1.00 |
| 1.75 | 1.00 | 1.00 | 1.00 | 1.00 | 1.00 | 1.00 | 1.00 | 1.00 | 1.00 |
| 2.00 | 1.00 | 1.00 | 1.00 | 1.00 | 1.00 | 1.00 | 0.35 | 0.35 | 0.35 |
| 2.25 | 1.00 | 1.00 | 1.00 | 0.35 | 0.35 | 0.35 | 0.35 | 0.35 | 0.35 |
| 2.50 | 0.35 | 0.35 | 0.35 | 0.35 | 0.35 | 0.35 | 0.35 | 0.35 | 0.35 |
| 2.75 | 0.35 | 0.35 | 0.35 | 0.35 | 0.35 | 0.35 | 0.35 | 0.35 | 0.35 |
| 3.00 | 0.35 | 0.35 | 0.35 | 0.35 | 0.35 | 0.35 | 0.35 | 0.35 | 0.35 |

**C: Task 3**

| Lambda\alpha | 1.00 | 0.98 | 0.96 | 0.94 | 0.92 | 0.90 | 0.88 | 0.86 | 0.84 |
| --- | --- | --- | --- | --- | --- | --- | --- | --- | --- |
| 1.00 | 1.00 | 1.00 | 1.00 | 1.00 | 1.00 | 1.00 | 1.00 | 1.00 | 1.00 |
| 1.25 | 1.00 | 1.00 | 1.00 | 1.00 | 1.00 | 1.00 | 1.00 | 1.00 | 1.00 |
| 1.50 | 1.00 | 1.00 | 1.00 | 1.00 | 1.00 | 1.00 | 1.00 | 1.00 | 1.00 |
| 1.75 | 1.00 | 1.00 | 1.00 | 1.00 | 1.00 | 1.00 | 1.00 | 1.00 | 1.00 |
| 2.00 | 1.00 | 1.00 | 1.00 | 1.00 | 1.00 | 1.00 | 1.00 | 1.00 | 1.00 |
| 2.25 | 1.00 | 1.00 | 1.00 | 1.00 | 1.00 | 1.00 | 1.00 | 1.00 | 1.00 |
| 2.50 | 1.00 | 1.00 | 1.00 | 1.00 | 1.00 | 1.00 | 1.00 | 1.00 | 1.00 |
| 2.75 | 1.00 | 1.00 | 1.00 | 1.00 | 1.00 | 1.00 | 1.00 | 1.00 | 1.00 |
| 3.00 | 1.00 | 1.00 | 1.00 | 1.00 | 1.00 | 1.00 | 1.00 | 1.00 | 0.37 |

**Table AII**

Optimal Investment Proportion in the Stock in the three tasks for PT Preferences with Reference Point at the Current Wealth ($W_{0}$) and with CPT decision weights, $\gamma=0.61, \delta=0.69$.

**A: Task 1**

| Lambda\alpha | 1.00 | 0.98 | 0.96 | 0.94 | 0.92 | 0.90 | 0.88 | 0.86 | 0.84 |
| --- | --- | --- | --- | --- | --- | --- | --- | --- | --- |
| 1.00 | 1.00 | 1.00 | 1.00 | 1.00 | 1.00 | 1.00 | 1.00 | 1.00 | 1.00 |
| 1.25 | 1.00 | 1.00 | 1.00 | 1.00 | 1.00 | 1.00 | 0.31 | 0.30 | 0.28 |
| 1.50 | 1.00 | 0.33 | 0.33 | 0.33 | 0.33 | 0.32 | 0.31 | 0.30 | 0.28 |
| 1.75 | 0.33 | 0.33 | 0.33 | 0.33 | 0.33 | 0.32 | 0.31 | 0.30 | 0.28 |
| 2.00 | 0.33 | 0.33 | 0.33 | 0.33 | 0.33 | 0.32 | 0.31 | 0.30 | 0.28 |
| 2.25 | 0.33 | 0.33 | 0.33 | 0.33 | 0.33 | 0.32 | 0.31 | 0.30 | 0.28 |
| 2.50 | 0.33 | 0.33 | 0.33 | 0.33 | 0.33 | 0.32 | 0.31 | 0.30 | 0.28 |
| 2.75 | 0.33 | 0.33 | 0.33 | 0.33 | 0.33 | 0.32 | 0.31 | 0.30 | 0.28 |
| 3.00 | 0.33 | 0.33 | 0.33 | 0.33 | 0.33 | 0.32 | 0.31 | 0.30 | 0.28 |

**B: Task 2**

| Lambda\alpha | 1.00 | 0.98 | 0.96 | 0.94 | 0.92 | 0.90 | 0.88 | 0.86 | 0.84 |
| --- | --- | --- | --- | --- | --- | --- | --- | --- | --- |
| 1.00 | 1.00 | 1.00 | 1.00 | 1.00 | 1.00 | 1.00 | 1.00 | 1.00 | 1.00 |
| 1.25 | 1.00 | 1.00 | 1.00 | 1.00 | 1.00 | 1.00 | 1.00 | 1.00 | 1.00 |
| 1.50 | 1.00 | 1.00 | 1.00 | 1.00 | 1.00 | 1.00 | 1.00 | 1.00 | 1.00 |
| 1.75 | 1.00 | 1.00 | 1.00 | 1.00 | 1.00 | 1.00 | 0.35 | 0.35 | 0.34 |
| 2.00 | 1.00 | 1.00 | 1.00 | 0.35 | 0.35 | 0.35 | 0.35 | 0.35 | 0.34 |
| 2.25 | 0.35 | 0.35 | 0.35 | 0.35 | 0.35 | 0.35 | 0.35 | 0.35 | 0.34 |
| 2.50 | 0.35 | 0.35 | 0.35 | 0.35 | 0.35 | 0.35 | 0.35 | 0.35 | 0.34 |
| 2.75 | 0.35 | 0.35 | 0.35 | 0.35 | 0.35 | 0.35 | 0.35 | 0.35 | 0.34 |
| 3.00 | 0.35 | 0.35 | 0.35 | 0.35 | 0.35 | 0.35 | 0.35 | 0.35 | 0.34 |

**C: Task 3**

| Lambda\alpha | 1.00 | 0.98 | 0.96 | 0.94 | 0.92 | 0.90 | 0.88 | 0.86 | 0.84 |
| --- | --- | --- | --- | --- | --- | --- | --- | --- | --- |
| 1.00 | 1.00 | 1.00 | 1.00 | 1.00 | 1.00 | 1.00 | 1.00 | 1.00 | 1.00 |
| 1.25 | 1.00 | 1.00 | 1.00 | 1.00 | 1.00 | 1.00 | 1.00 | 1.00 | 1.00 |
| 1.50 | 1.00 | 1.00 | 1.00 | 1.00 | 1.00 | 1.00 | 1.00 | 1.00 | 1.00 |
| 1.75 | 1.00 | 1.00 | 1.00 | 1.00 | 1.00 | 1.00 | 1.00 | 1.00 | 1.00 |
| 2.00 | 1.00 | 1.00 | 1.00 | 1.00 | 1.00 | 1.00 | 1.00 | 1.00 | 1.00 |
| 2.25 | 1.00 | 1.00 | 1.00 | 1.00 | 1.00 | 1.00 | 1.00 | 1.00 | 0.37 |
| 2.50 | 1.00 | 1.00 | 1.00 | 1.00 | 1.00 | 0.37 | 0.37 | 0.37 | 0.37 |
| 2.75 | 1.00 | 1.00 | 1.00 | 0.37 | 0.37 | 0.37 | 0.37 | 0.37 | 0.37 |
| 3.00 | 1.00 | 1.00 | 0.37 | 0.37 | 0.37 | 0.37 | 0.37 | 0.37 | 0.37 |

**Case 3: Reference point at the Expected Value of Wealth**

In this case the reference point depends on the return distributions under consideration, and on the subject's asset allocation choice. If the investor invests a proportion *w* in the stock, then his terminal wealth is:

$\tilde{W}_{T}=W_{0}\left[ w\tilde{R}+\left( 1-w \right)R_{f} \right]$, and the expected value of terminal wealth is:

$\bar{W}_{T}=W_{0}\left[ w\bar{R}+\left( 1-w \right)R_{f} \right]$ . The change of wealth relative to this reference point is:

$\tilde{x}\equiv\tilde{W}_{T}-\bar{W}_{T}=W_{0}w\left[ \tilde{R}-\bar{R} \right]$. (A15)

Denoting the deviation of the stock’s return from its average return by $\tilde{z}\equiv\tilde{R}-\bar{R}$ , the PT expected value is given by:

$EV\left( w \right)=W_{0}^{\alpha}w^{\alpha}\left[ -\lambda\int_{-\infty}^{0} f\left( z \right)\left( -z \right)^{\alpha}dz + \int_{0}^{\infty} f\left( z \right)z^{\alpha}dz \right]$, (A16)

where $f\left( z \right)$ is the probability density function of $z$. Similar to Case 1, again, this implies a corner solution for the optimal asset allocation, depending on the sign of the square brackets in eq.(25): if the sign is positive the optimal investment weight in the risky asset is $w*=1$, and if the sign is negative we have $w*=0$. Specifically, the optimal asset allocation is given by:

$w^{*} = \left\{ \begin{aligned} 0 if \lambda>\frac{\int_{0}^{\infty} f\left( z \right)z^{\alpha}dz}{\int_{-\infty}^{0} f\left( z \right)\left( -z \right)^{\alpha}dz} \equiv B \\ \\ \\ 1 if \lambda<\frac{\int_{0}^{\infty} f\left( z \right)z^{\alpha}dz}{\int_{-\infty}^{0} f\left( z \right)\left( -z \right)^{\alpha}dz} \equiv B \end{aligned} \right.$ . (A17)

Exactly as in Case 1, where the reference point is $W_{0}R_{f}$, here too we find a discontinuous jump in the optimal asset allocation from 0 to 1 as the return distribution changes. Notice that if the return distribution $\tilde{R}$ is symmetric, then the distribution of $\tilde{z}$, the distance of the return from the mean return, is symmetric around 0. In this case, the term *B* in eq.(A17) is equal to 1, and thus the optimal allocation to the stock is 0 for any PT investor with $\lambda\geq1$. Thus, in Task 1, which has a symmetric return distribution, the optimal allocation to the stock is 0. In Tasks 2 and 3 we also have *B*<1, and thus, again, the optimal allocation to the stock is 0 for any loss-averse PT investor.[[5]](#footnote-5) Thus, the prediction of PT with a reference point at the expected value of wealth is an allocation of 0 to the stock in all three tasks of our experiment.[[6]](#footnote-6)

**Case 4: Stochastic Reference point at the Future Value of Wealth Invested in the Stock**

Many funds managers are evaluated relative to a benchmark or index (Sharpe 1992). Some individual investors may also consider their reference point as the alternative of investing in the risky stock. This has led De Giorgi and Post (2011) to suggest a state-dependent stochastic reference point, which is the wealth if invested in the stock. This stochastic reference point also implies a corner solution for the optimal asset allocation, and a discontinuous jump for 0 to 1 in the optimal allocation to the stock, as shown below.

The investor’s reference point is his terminal wealth if invested in the stock, i.e. $W_{0}\tilde{R}$. If the investor invests a proportion *w* in the stock, then his terminal wealth is:

$\tilde{W}_{T}=W_{0}\left[ w\tilde{R}+\left( 1-w \right)R_{f} \right]$, and the change of wealth relative to the stochastic reference point is:

$\tilde{x}\equiv\tilde{W}_{T}-W_{0}\tilde{R}=W_{0}\left( 1-w \right)\left[ -\left( \tilde{R}-R_{f} \right) \right]$. (A18)

Denoting the stock return in *excess* of the risk-free rate by , we have: $\tilde{x}=W_{0}\left( 1-w \right)\left( -\tilde{r} \right)$ , and the PT expected value is given by:

$EV\left( w \right)=W_{0}^{\alpha}\left( 1-w \right)^{\alpha}\left[ -\lambda\int_{0}^{\infty} f\left( r \right)r^{\alpha}dr +\int_{-\infty}^{0} f\left( r \right)\left( -r \right)^{\alpha}dr \right]$. (A19)

(Notice that when $r$ is positive $x$ is negative, i.e. this is a loss. This is the reason that the integral over the positive values of $r$ are multiplied by the loss aversion coefficient $\lambda$). Similar to the cases analyzed in the text, this again implies a corner solution for the optimal asset allocation, depending on the sign of the square brackets in eq.(A19): if the sign is positive the optimal investment weight in the risky asset is $w*=0$, and if the sign is negative we have $w*=1$. Specifically, the optimal asset allocation is given by:

$w^{*} = \left\{ \begin{aligned} 0 if \lambda<\frac{\int_{-\infty}^{0} f\left( r \right)\left( -r \right)^{\alpha}dr}{\int_{0}^{\infty} f\left( r \right)r^{\alpha}dr}=\frac{1}{A} \\ \\ \\ 1 if \lambda>\frac{\int_{-\infty}^{0} f\left( r \right)\left( -r \right)^{\alpha}dr}{\int_{0}^{\infty} f\left( r \right)r^{\alpha}dr}=\frac{1}{A} \end{aligned} \right.$ . (A20)

Eq.(A20) implies a discontinuous jump in the optimal asset allocation from 0 to 1 as the return distribution changes, just like in Case 1. The intuition for eq.(A20) is the following: the investor dislikes deviations from the stock, which is his reference point, so a large $\lambda$ induces allocation of 1 to the stock. The investor is willing to deviate from holding the stock only if i) his $\lambda$ is not very large, and ii) the performance of the stock relative to the risk-free asset is not so good, i.e. the fraction is eq.(A20) is large. Notice that the fraction in eq.(A20) is exactly the inverse of the fraction in eq.(5) given in the text, which we denoted by *A*. The conditions (5) and (A20) are not identical: while eq.(5) implies that the optimal allocation to the stock is 100% if $\lambda<A$ , eq.(A20) implies this result for $\lambda>1/A$ . While these are different conditions, they do imply the same qualitative behavior: as the horizon increases, and the probability that the stock yields a higher return than the risk-free asset approaches 1, *A* increases indefinitely, and we have both $\lambda<A$ and $\lambda>1/A$, i.e. both cases imply an allocation of 100% to the stock. However, the jump to the stock may occur at different horizons under the two setups with alternative reference points.

1. Notice that as the limits of the integrals in eq.(A4) are also functions of *w*, one should employ the Leibnitz integral rule. In our specific case this does not change the value of the derivative, because the value of the function integrated at the integral limit which depends on *w* is zero. Let us elaborate. The Leibnitz rule is:

   $\frac{\partial}{\partial w}\left(\int_{a(w)}^{b(w)} g(w,r)dr\right)=g(w,b(w))\frac{\partial b(w)}{\partial w}-g(w,a(w))\frac{\partial a(w)}{\partial w}+\int_{a(w)}^{b(w)} \frac{\partial g(w,r)}{\partial w}dr$.

   If we take, for example, the derivative of the first term in eq.(A4), we have $g(w,r)\equiv\left(-\left(wr+r_{f}\right)\right)^{\alpha}f(r)$, and therefore:

   $\begin{matrix} \frac{\partial}{\partial w}\left(\int_{r_{\min}}^{-\frac{r_{f}}{w}} \left(-\left(wr+r_{f}\right)\right)^{\alpha}f(r) dr\right)=\left(-\left(w\left(-\frac{r_{f}}{w}\right)+r_{f}\right)\right)^{\alpha}f(-\frac{r_{f}}{w})\frac{\partial}{\partial w}\left(-\frac{r_{f}}{w}\right)-\left(-\left(wr{}_{\min}+r_{f}\right)\right)^{\alpha}f(r{}_{\min})\frac{\partial}{\partial w}\left(r_{\min}\right)+ \\ \alpha\int_{r_{\min}}^{-\frac{r_{f}}{w}} \left(-\left(wr+r_{f}\right)\right)^{\alpha-1}\left(-r\right)f(r) dr. \end{matrix}$The first term is equal to 0 because the value of the function *g* at the upper limit is 0, and the second term is 0 because the lower limit does not depend on *w*. Thus, we are left with only the third term. Similarly, in the derivative of the second term in eq.(A4), the first term is 0 because the upper limit does not depend on *w*, and the second term is 0 because the value of the function *g* at the lower limit is 0, and again, we are left with only the third term, which is $\alpha\int_{-\frac{r_{f}}{w}}^{\infty} \left(wr+r_{f}\right)^{\alpha-1}r f(r) dr$. [↑](#footnote-ref-1)
2. Actually, if borrowing at the risk-free rate is possible (which is not the setup of our experiment) and the reference point is at the current wealth, as the horizon increases the optimal investment proportion in the stock becomes infinite. To see this, it is helpful to analyze the derivative in eq.(A7) in the case $w> >r_{f}$. Note that eq.(A7) can be rewritten as:

   $\frac{\partial EV}{\partial w}=\alpha w^{\alpha-1}\left[-\lambda\int_{r_{\min}}^{-\frac{r_{f}}{w}} \left(-\left(r+\frac{r_{f}}{w}\right)\right)^{\alpha-1}\left(-r\right)f(r) dr +\int_{-\frac{r_{f}}{w}}^{0} \left(r+\frac{r_{f}}{w}\right)^{\alpha-1}r f(r) dr+\int_{0}^{\infty} \left(r+\frac{r_{f}}{w}\right)^{\alpha-1}r f(r) dr\right]$.

   When $w> >r_{f}$, this expression reduces to:

   $\frac{\partial EV}{\partial w}\cong\alpha w^{\alpha-1}\left[-\lambda\int_{r_{\min}}^{0} \left(-r\right)^{\alpha}f(r) dr +\int_{0}^{\infty} r ^{\alpha}f(r) dr\right]$. By integration over *w*, this implies that for large *w* we have $EV\cong w^{\alpha}\left[-\lambda\int_{r_{\min}}^{0} \left(-r\right)^{\alpha}f(r) dr +\int_{0}^{\infty} r ^{\alpha}f(r) dr\right]$, just like in the case of a reference point at the future value of wealth (see eq.(4)). As the horizon increases, the probability of a negative excess return shrinks, and for any $\lambda$ there comes a horizon where $\lambda<\int_{0}^{\infty} f(r) r^{\alpha}dr/\int_{-\infty}^{0} f(r)(-r)^{\alpha}dr$ , i.e. the square brackets become positive. This implies that for large *w* the derivative is positive and constant, i.e. it is optimal to borrow at the risk-free rate and invest an infinite amount in the stock. Note that the convergence to the case of a reference point at the future value of wealth holds for $w> >r_{f}$. For lower values of *w*, the optimal asset allocation is different when the different reference points are employed. [↑](#footnote-ref-2)
3. Equating the derivative in eq.(A12) to zero yields: $\frac{0.05+0.25w^{*}}{0.05-0.15w^{*}}=\left(\frac{0.15}{0.25}\right)^{\frac{1}{\alpha-1}}=\left(\frac{3}{5}\right)^{\frac{1}{\alpha-1}}$. Note that for $\alpha\to1$ we obtain *w**=1/3 as a special case of eq.(A13). [↑](#footnote-ref-3)
4. In the range *w*>1/3 the derivative is:

   $\frac{\partial EV}{\partial w}=\frac{1}{2}\left[0.25\alpha\left(0.25w+0.05\right)^{\alpha-1}-0.15\lambda\alpha\left(0.15w-0.05\right)^{\alpha-1}\right]$. Equating this derivative to zero (in search for a maximum point) yields: $w*=\frac{0.05\cdot\left(\frac{3}{5}\lambda\right)^{\frac{1}{\alpha-1}} + 0.05}{0.15\cdot\left(\frac{3}{5}\lambda\right)^{\frac{1}{\alpha-1}} - 0.25}$. A little algebra reveals that this expression is either negative (if $\lambda>\left(\frac{5}{3}\right)^{\alpha}$) or larger than 1 (if $\lambda<\left(\frac{5}{3}\right)^{\alpha}$). Thus, there is no maximum (or minimum) point in the range 1>*w*>1/3. Note that for $w>>0.05$the *EV* becomes $EV=\frac{1}{2}w^{\alpha}\left[0.25^{\alpha}-\lambda0.15^{\alpha}\right]$, and continuous to grow (or decline) indefinitely with *w*. [↑](#footnote-ref-4)
5. For example, in Task 2 we have $\bar{R}=1.21$ ,

   and $\tilde{z}=(-0.4 with prob. 0.25; -0.04 with prob. 0.5; 0.48 with prob. 0.25;)$. This implies

   $B=\frac{0.25\cdot{0.48}^{\alpha}}{0.5\cdot{0.04}^{\alpha}+ 0.25\cdot{0.4}^{\alpha}}$, or $=\frac{{0.48}^{\alpha}}{2\cdot{0.04}^{\alpha}+ {0.4}^{\alpha}}$ , which is smaller than 1 for any $\alpha<1$. Similarly, in Task 3 we have $\bar{R}=1.331$ and $=\frac{\frac{3}{8}{0.190}^{\alpha}+\frac{1}{8}{0.866}^{\alpha}}{\frac{3}{8}{0.278}^{\alpha}+\frac{1}{8}{0.602}^{\alpha}}$ , which is, again, smaller than 1 for any $\alpha<1$. [↑](#footnote-ref-5)
6. The setup with reference point at the expected value of wealth typically implies an optimal allocation of 1 to the stock when the return distribution is negatively skewed. For example, if the rate of return on the stock is -50% with probability 0.1, and 20% with probability 0.9, we have $\bar{R}=1.13$ and $B=\frac{0.9\cdot{0.07}^{\alpha}}{0.1\cdot{0.63}^{\alpha}}$. For $\alpha=0.8$ , for example, we have $B=1.55$, and thus for any PT investor with $\lambda<1.55$ the optimal investment proportion in the stock is 1. [↑](#footnote-ref-6)
