# Supplementary material for: Prospect theory, constant relative risk aversion, and the investment horizon
Source: PLoS One. 2021 Apr 1;16(4):e0248904. doi: 10.1371/journal.pone.0248904 (PMC8016345; doi:10.1371/journal.pone.0248904)
Supplement: S3 Appendix — (DOCX) [file pone.0248904.s003.docx]

**S3 Appendix**

**
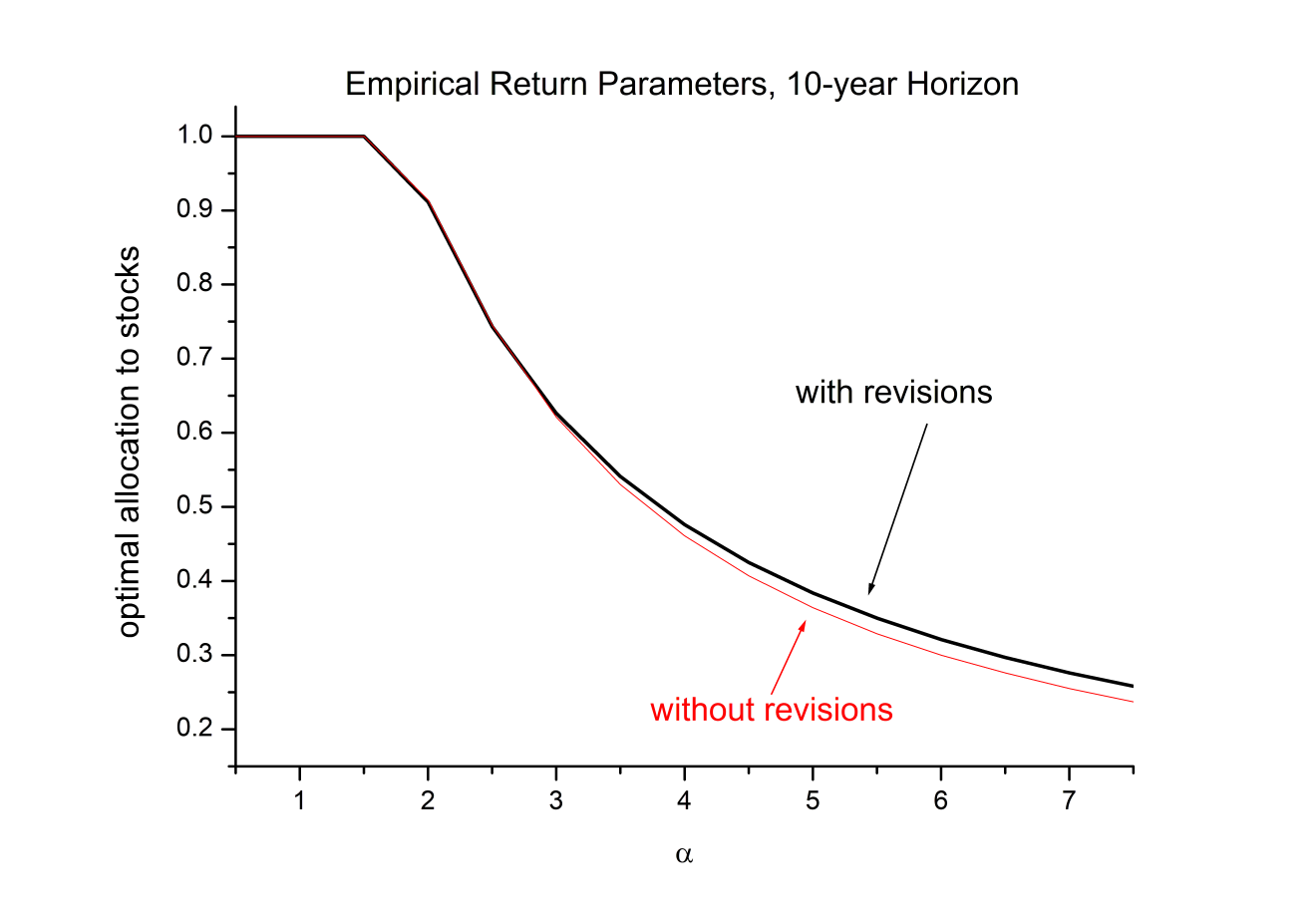
**

The optimal asset allocation between the S&P500 index and 3-month T-bills for CRRA investors with a 10-year investment horizon. The optimal allocation is calculated when the portfolio can be revised annually (bold line), and when no revisions are possible (thin line). We employ the empirical returns during 1997-2016. For the 10-year horizon, the 10-year return distributions are estimated by randomly drawing 50,000 sets of 10 returns (with replacement) from the annual returns on the S&P500 index during 1997-2016. The T-bill rate is taken as the average annual rate over this period (2.14%). The figure shows that the optimal asset allocation is almost the same whether revisions are allowed or not. Thus, a CRRA investor should optimally allocate approximately the same proportion to stocks whether he invests for one year or for ten years, even if portfolio revisions are not possible.
