# Supplementary material for: Prospect theory, constant relative risk aversion, and the investment horizon
Source: PLoS One. 2021 Apr 1;16(4):e0248904. doi: 10.1371/journal.pone.0248904 (PMC8016345; doi:10.1371/journal.pone.0248904)
Supplement: S4 Appendix — (DOCX) [file pone.0248904.s004.docx]

**S4 Appendix: The Questionnaire**

Below is the questionnaire used in the experiment, Tasks 1-3 are the main tasks, corresponding to horizons of 1-3 periods, respectively. Task 4 is the control task: Investment G dominates investment F by First-degree Stochastic Dominance (FSD). Thus, we expect all rational subjects to choose G. This task is used to verify that the subjects understood the setup and paid careful attention to the experiment. Indeed, 90% of the subjects chose G. The results reported in Tasks 1-3 are only for these 90% (but they do not change much if all subjects are included).

**Investment Choice Questionnaire**

This is an investment choice questionnaire, composed of 4 investment tasks. The questionnaire is anonymous, and will be used for academic research purposes only. Participation is voluntary. Please consider these tasks carefully, and answer according to *your* preference. Thank you!

**Task 1:**

*Suppose that you have decided to invest $100,000 either in a stock or in a risk-free bond or in any combination of these two assets. The possible outcomes at the end of the investment period are as given below. Please write the percentage of the $100,000 you choose to invest in each asset, where the sum of the two investment proportions should add up to 100%.*

*Investment proportion in stock: ______ Investment proportion in risk-free bond: ______*

| **If you invest 100% in the stock** | | |  | **If you invest 100% in the risk-free bond** | | |
| --- | --- | --- | --- | --- | --- | --- |
| *Outcome* | *Rate of Return* | *Probability* |  | *Outcome* | *Rate of Return* | *Probability* |
| $90,000 | -10% | 50% |  | $105,000 | 5% | 100% |
| $130,000 | 30% | 50% |  |  |  |  |

______________________________________________________________________________

**Task 2:**

*Suppose that you have decided to invest $100,000 either in a stock or in a risk-free bond or in any combination of these two assets. The possible outcomes at the end of the investment period are as given below. Please write the percentage of the $100,000 you choose to invest in each asset, where the sum of the two investment proportions should add up to 100%.*

*Investment proportion in stock: ______ Investment proportion in risk-free bond: ______*

| **If you invest 100% in the stock** | | |  | **If you invest 100% in the risk-free bond** | | |
| --- | --- | --- | --- | --- | --- | --- |
| *Outcome* | *Rate of Return* | *Probability* |  | *Outcome* | *Rate of Return* | *Probability* |
| $81,000 | -19% | 25% |  | $110,250 | 10.25% | 100% |
| $117,000 | 17% | 50% |  |  |  |  |
| $169,000 | 69% | 25% |  |  |  |  |

______________________________________________________________________________

**Task 3:**

*Suppose that you have decided to invest $100,000 either in a stock or in a risk-free bond or in any combination of these two assets. The possible outcomes at the end of the investment period are as given below. Please write the percentage of the $100,000 you choose to invest in each asset, where the sum of the two investment proportions should add up to 100%.*

*Investment proportion in stock: ______ Investment proportion in risk-free bond: ______*

| **If you invest 100% in the stock** | | |  | **If you invest 100% in the risk-free bond** | | |
| --- | --- | --- | --- | --- | --- | --- |
| *Outcome* | *Rate of Return* | *Probability* |  | *Outcome* | *Rate of Return* | *Probability* |
| $72,900 | -27.1% | 12.5% |  | $115,762 | 15.76% | 100% |
| $105,300 | 5.3% | 37.5% |  |  |  |  |
| $152,100 | 52.1% | 37.5% |  |  |  |  |
| $219,700 | 119.7% | 12.5% |  |  |  |  |

______________________________________________________________________________

**Task 4:**

Suppose that you can invest the $100,000 either in investment F or in investment G (but you cannot diversify between the two investments) with the following end of period values:

**Investment F Investment G**

*Outcome Probability Outcome Probability*

$110,000 50% $110,000 25%

$120,000 50% $120,000 50%

$130,000 25%

Which investment will you choose? _________ (Please write F or G)
