# Supplementary material for: Prospect theory, constant relative risk aversion, and the investment horizon
Source: PLoS One. 2021 Apr 1;16(4):e0248904. doi: 10.1371/journal.pone.0248904 (PMC8016345; doi:10.1371/journal.pone.0248904)
Supplement: S5 Appendix — (DOCX) [file pone.0248904.s005.docx]

**S5 Appendix: The Average Allocation to the Stock, by Group and by Task**

The average asset allocations in a given task are similar across the four subject groups. The differences between groups are not statistically significant. Specifically, the null hypothesis of homogenous investment choices of the four groups in Task 1, Task 2 and Task 3 cannot be rejected (p= 51.9%, 45.2% and 7.7% , respectively). When we regress the individual investment proportions in a given task on group dummy variables none of the dummy coefficients are significant.

| Group | | Tasks | | | Average across tasks |
| --- | --- | --- | --- | --- | --- |
|  |  | Task 1 | Task 2 | Task 3 |  |
| 1 | China (undergraduate students) | 52.4% | 55.2% | 54.8% | 54.1% |
| 2 | Hong Kong –  (Master students) | 46.7% | 55.4% | 63.4% | 55.2% |
| 3 | Israel –  (Master students) | 58.8% | 63.8% | 66.0% | 62.9% |
| 4 | Israel – (Professional investors) | 52.2% | 56.9% | 52.8% | 54.0% |
|  | Average across groups | 53.7% | 59.0% | 58.1% | 56.7% |

**Individual-Level Data**

The table below reports the individual-level data for all 182 subjects who completed the questionnaire and answered the control task (Task 4) correctly. The allocation to the stock in Tasks 1-3 is given in percent.

| **Subject number** | **Group** | **Age** | **Sex** | **Task1** | **Task2** | **Task3** |
| --- | --- | --- | --- | --- | --- | --- |
| 1 | 1 | 25 | M | 50 | 100 | 100 |
| 2 | 1 | 25 | M | 40 | 60 | 80 |
| 3 | 1 | 22 | F | 100 | 50 | 37.5 |
| 4 | 1 | 21 | F | 100 | 100 | 100 |
| 5 | 1 | 24 | F | 40 | 50 | 75 |
| 6 | 1 | 22 | F | 100 | 100 | 50 |
| 7 | 1 | 22 | F | 100 | 100 | 50 |
| 8 | 1 | 24 | F | 50 | 50 | 40 |
| 9 | 1 | 25 | F | 70 | 60 | 50 |
| 10 | 1 | 21 | F | 100 | 100 | 100 |
| 11 | 1 | 24 | F | 50 | 50 | 50 |
| 12 | 1 | 25 | F | 30 | 40 | 50 |
| 13 | 1 | 25 | F | 35 | 40 | 50 |
| 14 | 1 | 25 | F | 50 | 45 | 50 |
| 15 | 1 | 24 | F | 10 | 30 | 5 |
| 16 | 1 | 25 | F | 100 | 100 | 100 |
| 17 | 1 | 25 | F | 30 | 30 | 30 |
| 18 | 1 | 23 | F | 0 | 50 | 50 |
| 19 | 1 | 25 | F | 40 | 30 | 20 |
| 20 | 1 | 24 | F | 100 | 100 | 100 |
| 21 | 1 | 25 | F | 20 | 20 | 20 |
| 22 | 1 | 24 | F | 10 | 10 | 10 |
| 23 | 1 | 25 | F | 30 | 30 | 30 |
| 24 | 1 | 24 | F | 40 | 40 | 40 |
| 25 | 1 | 25 | F | 50 | 50 | 50 |
| 26 | 1 | 24 | F | 40 | 40 | 40 |
| 27 | 1 | 24 | F | 50 | 50 | 50 |
| 28 | 1 | 25 | F | 60 | 60 | 60 |
| 29 | 1 | 24 | F | 45 | 45 | 45 |
| 30 | 1 | 25 | F | 50 | 50 | 50 |
| 31 | 1 | 25 | F | 60 | 60 | 60 |
| 32 | 1 | 24 | F | 20 | 20 | 20 |
| 33 | 1 | 24 | F | 50 | 50 | 50 |
| 34 | 1 | 25 | F | 50 | 50 | 50 |
| 35 | 1 | 25 | F | 10 | 10 | 10 |
| 36 | 1 | 24 | F | 0 | 50 | 60 |
| 37 | 1 | 25 | F | 100 | 0 | 100 |
| 38 | 1 | 26 | M | 70 | 80 | 55 |
| 39 | 1 | 25 | M | 100 | 100 | 100 |
| 40 | 1 | 30 | M | 100 | 100 | 0 |
| 41 | 1 | 23 | M | 100 | 100 | 100 |
| 42 | 1 | 24 | M | 60 | 40 | 50 |
| 43 | 1 | 23 | M | 40 | 100 | 90 |
| 44 | 1 | 24 | M | 20 | 30 | 40 |
| 45 | 1 | 28 | M | 50 | 70 | 90 |
| 46 | 1 | 25 | M | 100 | 0 | 100 |
| 47 | 1 | 30 | M | 80 | 90 | 95 |
| 48 | 1 | 24 | M | 0 | 80 | 50 |
| 49 | 1 | 25 | M | 20 | 80 | 80 |
| 50 | 1 | 24 | M | 30 | 30 | 30 |
| 51 | 1 | 24 | M | 40 | 40 | 40 |
| 52 | 1 | 24 | M | 50 | 50 | 50 |
| 53 | 1 | 24 | M | 50 | 50 | 50 |
| 54 | 1 |  |  | 50 | 40 | 30 |
| 55 | 1 |  |  | 40 | 35 | 30 |
| 56 | 2 | 25 | F | 70 | 80 | 90 |
| 57 | 2 | 26 | F | 60 | 75 | 80 |
| 58 | 2 | 27 | F | 65 | 75 | 80 |
| 59 | 2 | 29 | F | 30 | 50 | 60 |
| 60 | 2 | 42 | F | 0 | 100 | 100 |
| 61 | 2 | 25 | M | 33.3 | 34.5 | 36.8 |
| 62 | 2 | 27 | M | 75 | 75 | 85 |
| 63 | 2 | 26 | M | 50 | 0 | 0 |
| 64 | 2 | 26 | M | 90 | 80 | 70 |
| 65 | 2 | 27 | M | 0.0 | 33.3 | 33.3 |
| 66 | 2 | 28 | M | 33.3 | 50.0 | 66.7 |
| 67 | 2 | 31 | M | 70.0 | 70.0 | 70.0 |
| 68 | 2 | 28 | M | 33.3 | 33.3 | 100.0 |
| 69 | 2 | 30 | M | 40 | 45 | 50 |
| 70 | 2 | 26 | M | 50 | 30 | 30 |
| 71 | 3 |  |  | 100 | 100 | 50 |
| 72 | 3 |  |  | 10 | 90 | 60 |
| 73 | 3 |  |  | 75 | 70 | 65 |
| 74 | 3 | 29 | F | 100 | 100 | 100 |
| 75 | 3 | 26 | F | 20 | 30 | 30 |
| 76 | 3 | 26 | F | 100 | 75 | 25 |
| 77 | 3 | 30 | F | 70 | 70 | 70 |
| 78 | 3 | 30 | F | 50 | 30 | 30 |
| 79 | 3 | 30 | F | 100 | 70 | 50 |
| 80 | 3 | 23 | F | 33 | 34.5 | 36.7 |
| 81 | 3 | 27 | F | 30 | 70 | 85 |
| 82 | 3 | 27 | F | 20 | 70 | 80 |
| 83 | 3 | 32 | F | 70 | 90 | 90 |
| 84 | 3 | 50 | F | 40 | 75 | 60 |
| 85 | 3 | 21 | M | 80 | 80 | 75 |
| 86 | 3 | 25 | M | 60 | 65 | 70 |
| 87 | 3 | 25 | M | 50 | 25 | 70 |
| 88 | 3 | 28 | M | 20 | 25 | 20 |
| 89 | 3 | 31 | M | 100 | 100 | 100 |
| 90 | 3 | 25 | M | 30 | 30 | 30 |
| 91 | 3 | 26 | M | 80 | 70 | 70 |
| 92 | 3 | 26 | M | 66.7 | 66.7 | 50.0 |
| 93 | 3 | 34 | M | 75 | 75 | 90 |
| 94 | 3 | 25 | M | 100 | 100 | 100 |
| 95 | 3 | 29 | M | 100 | 100 | 100 |
| 96 | 3 | 27 | M | 75 | 100 | 100 |
| 97 | 3 | 39 | M | 30 | 50 | 70 |
| 98 | 3 | 31 | M | 0 | 0 | 0 |
| 99 | 3 | 27 | M | 30 | 30 | 30 |
| 100 | 3 | 28 | M | 20 | 20 | 25 |
| 101 | 3 | 28 | M | 0 | 30 | 70 |
| 102 | 3 | 37 | M | 50 | 75 | 100 |
| 103 | 3 | 34 | M | 50 | 70 | 50 |
| 104 | 3 | 29 | M | 50 | 70 | 80 |
| 105 | 3 | 29 | M | 100 | 100 | 80 |
| 106 | 3 | 29 | M | 50 | 50 | 40 |
| 107 | 3 | 27 | M | 0 | 35 | 100 |
| 108 | 3 | 29 | M | 80 | 90 | 95 |
| 109 | 3 | 42 | M | 50 | 80 | 80 |
| 110 | 3 | 27 | M | 40 | 80 | 100 |
| 111 | 3 | 32 | M | 100 | 75 | 25 |
| 112 | 3 | 29 | M | 70 | 90 | 80 |
| 113 | 3 | 30 | M | 100 | 80 | 70 |
| 114 | 3 | 38 | M | 100 | 50 | 70 |
| 115 | 3 | 28 | M | 100 | 100 | 70 |
| 116 | 3 | 36 | M | 20 | 70 | 90 |
| 117 | 3 | 32 | M | 80 | 60 | 90 |
| 118 | 3 | 24 | M | 30 | 20 | 40 |
| 119 | 3 | 26 | M | 20 | 25 | 30 |
| 120 | 3 | 27 | M | 100 | 100 | 100 |
| 121 | 3 | 31 | M | 70 | 90 | 50 |
| 122 | 3 | 29 | M | 40 | 80 | 90 |
| 123 | 4 |  |  | 27 | 37 | 38 |
| 124 | 4 |  |  | 31 | 46.8 | 23.9 |
| 125 | 4 | 25 | F | 20 | 60 | 80 |
| 126 | 4 | 40 | F | 70 | 80 | 75 |
| 127 | 4 | 26 | M | 0 | 5 | 10 |
| 128 | 4 | 28 | M | 100 | 100 | 100 |
| 129 | 4 | 27 | M | 20 | 30 | 20 |
| 130 | 4 | 27 | M | 50 | 50 | 50 |
| 131 | 4 | 29 | M | 50 | 50 | 20 |
| 132 | 4 | 35 | M | 0 | 100 | 100 |
| 133 | 4 | 33 | M | 40 | 70 | 80 |
| 134 | 4 | 36 | M | 60 | 70 | 80 |
| 135 | 4 | 52 | M | 100 | 100 | 100 |
| 136 | 4 | 38 | M | 10 | 0 | 10 |
| 137 | 4 | 45 | M | 25 | 25 | 20 |
| 138 | 4 | 46 | M | 30 | 80 | 100 |
| 139 | 4 | 45 | M | 100 | 100 | 100 |
| 140 | 4 | 61 | M | 80 | 90 | 70 |
| 141 | 4 | 35 | M | 90 | 80 | 60 |
| 142 | 4 | 42 | M | 50 | 70 | 80 |
| 143 | 4 | 35 | F | 50 | 40 | 50 |
| 144 | 4 | 58 | F | 100 | 100 | 100 |
| 145 | 4 | 46 | F | 0 | 0 | 0 |
| 146 | 4 | 45 | F | 100 | 100 | 50 |
| 147 | 4 | 30 | M | 20 | 20 | 30 |
| 148 | 4 | 35 | M | 100 | 100 | 100 |
| 149 | 4 | 57 | M | 30 | 30 | 15 |
| 150 | 4 | 30 | M | 30 | 30 | 30 |
| 151 | 4 | 53 | M | 40 | 50 | 40 |
| 152 | 4 | 35 | M | 100 | 100 | 100 |
| 153 | 4 | 35 | M | 100 | 100 | 100 |
| 154 | 4 | 32 | M | 100 | 100 | 100 |
| 155 | 4 | 37 | M | 100 | 100 | 100 |
| 156 | 4 | 29 | M | 20 | 20 | 20 |
| 157 | 4 | 47 | M | 0 | 0 | 13.6 |
| 158 | 4 | 30 | M | 100 | 100 | 100 |
| 159 | 4 | 34 | M | 50 | 40 | 30 |
| 160 | 4 | 60 | M | 100 | 100 | 100 |
| 161 | 4 | 44 | M | 55 | 55 | 55 |
| 162 | 4 | 47 | M | 100 | 100 | 100 |
| 163 | 4 |  |  | 50 | 80 | 20 |
| 164 | 4 |  |  | 30 | 35 | 40 |
| 165 | 4 | 34 | F | 0 | 0 | 0 |
| 166 | 4 | 40 | F | 50 | 60 | 60 |
| 167 | 4 | 34 | F | 0 | 0 | 0 |
| 168 | 4 | 34 | F | 30 | 30 | 30 |
| 169 | 4 | 28 | M | 30 | 10 | 0 |
| 170 | 4 | 28 | M | 33 | 34 | 36 |
| 171 | 4 | 35 | M | 100 | 100 | 100 |
| 172 | 4 | 32 | M | 10 | 0 | 0 |
| 173 | 4 | 28 | M | 70 | 50 | 40 |
| 174 | 4 | 26 | M | 100 | 90 | 70 |
| 175 | 4 | 36 | M | 40 | 50 | 30 |
| 176 | 4 | 41 | M | 100 | 70 | 50 |
| 177 | 4 | 33 | M | 100 | 100 | 100 |
| 178 | 4 | 40 | M | 30 | 20 | 10 |
| 179 | 4 | 32 | M | 60 | 40 | 30 |
| 180 | 4 | 35 | M | 60 | 50 | 35 |
| 181 | 4 | 31 | M | 20 | 10 | 25 |
| 182 | 4 | 38 | M | 0 | 75 | 50 |
