# Supplementary material for: Prospect theory, constant relative risk aversion, and the investment horizon
Source: PLoS One. 2021 Apr 1;16(4):e0248904. doi: 10.1371/journal.pone.0248904 (PMC8016345; doi:10.1371/journal.pone.0248904)
Supplement: Attachment — Submitted filename: Revision Report.pdf [file pone.0248904.s006.pdf]

Revision Report - Prospect Theory, Constant Relative Risk Aversion, and the Investment Horizon

(PONE-D-21-00889)

Thank you very much for your thoughtful and constructive comments, they are greatly appreciated. We believe that your comments have helped us significantly improve the paper. Below we reproduce your comments in italics, followed by the revisions made in light of each comment.

The submitted paper can be divided in two sections. In the first part, the authors demonstrate 1) a CRRA implies an optimal allocation between a riskless asset and a risky stock independent of the time horizon; 2) a PT implies a "jump" in the optimal allocation increasing the horizon. In the second part, the authors search for experimental support on these two results finding that most of the agents is CRRA with only few subjects which behave consistently with PT (at most 6%). I found the paper unbalanced in the explanation of the two theoretical approaches: authors deeply describe the theoretical implications of PT, whereas section 3 (CRRA) is not well developed. Since PlosOne is a transdisciplinary journal, this lack of explanation is a real problem for the understanding of the readers.

Thank you for pointing this out. We revised the paper to be more balanced between the two parts, and the discussion of CRRA is now derived in much more detail (pages 16-21 of the revised version).

Some comments:

- *Pg. 2; authors wrote that the jump "does not depend on the return distribution, the specifics of the PT value function parameters, or on the use of the cumulative prospect theory". Can the authors provide some explanations on the parameters affecting the "jump" property?*

We added the following paragraph on pages 7-8 to address this issue:

> The optimal asset allocation given by eq.(5) depends on the preference parameters, λ and α, as well as on the excess return distribution $f(r)$. The excess return distribution and α determine the value of A. Thus, for a given return

distribution and α there is a threshold value of the loss aversion parameter λ, below which the allocation to the stock is 100%, and above which the allocation is 0%. Intuitively, the lower the loss aversion, the more attractive the stock seems. If the distribution of excess returns, $f(r)$, is "improved" in the sense that the probability of a loss decreases and the probability of a gain increases, the value of A typically increases (its denominator is decreased and its numerator is increased). This implies that the threshold λ below which the optimal allocation to the stock is 100% increases, implying that the stock becomes the preferred choice for a wider spectrum of PT investors. As will become evident in what follows, this is exactly what happens when the investment horizon increases. The effect of the power exponent α on choice is more ambiguous, as it affects both the numerator and the denominator of A. The effect of α on the optimal allocation may thus depend on the exact distribution of $f(r)$, and in particular, on it's a-symmetry. Indeed, numerical calculation of the optimal asset allocation, reported in Table II, reveals that the value of α has only a small effect on the optimal allocation.

- Pg. 6; If I have correctly understood, the excess of the risk-free rate is $\tilde{r}$, hence in equations (4)-(5) should be $\tilde{r}$ not r.

To avoid confusion we now define: $r \equiv R - R_f$.

- Pg. 10; It is not clear to me what happens in case of equality in equation (7).

We add the following on page 7 to address this case:

In the special case where $A = 0$ the expected value is independent of the asset allocation, implying that the investor is indifferent to the allocation (and is also indifferent between participation in the market and the status-quo of remaining with the initial wealth).

- Pg. 13; It is not clear to me how have been defined the range of the parameters. In these tables the authors allow for lambda equal to 1 but, in section 5, they state that lambda is greater than 1.75.

Indeed, in order to examine the robustness of the results, the tables cover parameter ranges that are wider than the typical experimental estimates. To clarify this point we added on page 13:

While the experimental estimates of λ are typically in the range 1.8-2.3 (see Brown et. al. 2021 for a recent meta-analysis) and α is typically estimated to be in the neighborhood of 0.9, Table II reports the optimal asset allocation for a much wider range of parameters. This allows us to examine the robustness of the results to the parameter values, and to address the issue of possible heterogeneity among PT investors (as the parameter estimates are only population averages).

- Pg. 15, authors state that the optimal allocation using CRRA is independent of the initial wealth. If I am not wrong, this also applies for PT.

We completely agree. We have added a statement to this effect on page 17 of the revised version.

- Pg. 19, authors write that Task-2 and Task-3 are the 2-period and 3-period version of Task-1 (one period) even if they are presented as three different stand-alone tasks. They justify this statement quoting Thaler et al. (1997) but, if I am not wrong, in that manuscript the subjects know the different horizon time (monthly, yearly, 5-yearly). - Related to this issue, it is not clear to me how the authors can investigate the change in the horizon presenting to the subject three stand-alone tasks without any reference on time. The three situations can be perceived as three completely different asset allocations due to the bounded rationality of the subjects.

Indeed, in Thaler et. al. (1997) subjects are told that they will make decisions either "every financial period" or "every eight financial periods", depending on the treatment (page 660 in Thaler et. al.). However, crucially, the subjects in the eight-period treatment only observe the eight-period returns, not the single-period returns (Thaler et. al. page 653). It is important that the subjects observe the return distribution that corresponds to their relevant horizon, otherwise biases may arise, as shown by Benartzi and Thaler (1999). To emphasize this point we add the following on page 22:

> The experimental setup employed corresponds to the framework in which the theoretical predictions the optimal asset allocation of PT and CRRA investors are derived – the optimal allocation is based on the t-period return distributions, which are the distributions presented to the subjects.

- Pg. 20, the authors should provide a deeper explanation on the fact that 1) the large hypothetical gains and losses may be perceived as more realistic; 2) the subjects act consistently with these possible large gains/losses, i.e. they make choices carefully, if there no incentives (as some payments) in performing accordingly (the authors only introduce a control task).

We rewrote the discussion on the pros and cons of the large-scale hypothetical setup of the experiment. In particular, we clarify and emphasize the role of the control task in verifying that the vast majority of subjects (90%) paid careful attention to the

experimental tasks, and in screening-out the few subjects who either did not give the tasks careful consideration, or did not fully understand the instructions (pages 22-23 of the revised version).

- *Pg. 21, the original questionnaire is not provided.*

Thank you. Now provided in Appendix D (Supplementary Material S4).

- *Pg. 22, the individual level data is not provided.*

Thank you. Now provided in Appendix E (Supplementary Material S5).

- *Pg. 28, Thaler et al. (1997) is not a one-shot decision, subjects made repeated decisions over different horizon.*

Indeed, we should have made note of this. In the revised version we now address the differences between the Thaler et. al. (1997) and Gneezy and Potters (1997) experiments, and their relation to our own setup (page 31):

> One difference is that TTKS consider the allocation between a stock and a *risky* bond, while GP consider a stock and a *risk-free* bond (as in our setup). Another difference is that in the TTKS setup subjects learn the return parameters by repeatedly observing return realizations, while in GP and in our experiment the return distributions are known to the subjects.

- *Pg. 32, "the second implication of our results is about the important role of heterogeneity. [...] subjects are very different [...] follow different patterns of the asset allocation". There is a huge literature on agent-based and asset price, and their experimental application, which is completely absent in the introduction.*

We add the following discussion of heterogeneity in the introduction (page 4):

> Analysis of the results at the individual level yields not only a sharp test of the competing theories, but also allows an examination of the degree of heterogeneity among individuals. While the concept of a "representative agent" is widely employed, numerous studies have shown that heterogeneity may have fundamental economic consequences (see, for example, Lucas 1976, Kirman 1992, 1993, Levy, Levy and Solomon 1994, Gatti, Gallegati, and Kirman 2012, and Blackburn and Ukhov 2013).
